# LRRC4 Suppresses E-Cadherin-Dependent Collective Cell Invasion and Metastasis in Epithelial Ovarian Cancer

**DOI:** 10.3389/fonc.2020.00144

**Published:** 2020-02-14

**Authors:** Chunhua Zhao, Xiaoling She, Yan Zhang, Changhong Liu, Peiyao Li, Shuai Chen, Buqing Sai, Yunchao Li, Jianbo Feng, Jia Liu, Yingnan Sun, Songshu Xiao, Liping Li, Minghua Wu

**Affiliations:** ^1^Hunan Provincial Tumor Hospital and the Affiliated Tumor Hospital of Xiangya Medical School, Central South University, Changsha, China; ^2^School of Basic Medical Science, Cancer Research Institute, Central South University, Changsha, China; ^3^Key Laboratory of Carcinogenesis and Cancer Invasion, Ministry of Education, Changsha, China; ^4^Key Laboratory of Carcinogenesis, Ministry of Health, Changsha, China; ^5^Sun Yat-sen University Cancer Center, State Key Laboratory of Oncology in South China, Guangzhou, China; ^6^Second Xiangya Hospital, Central South University, Changsha, China; ^7^Third Xiangya Hospital, Central South University, Changsha, China; ^8^The Affiliated Zhuzhou Hospital of XiangYa Medical School, Central South University, Changsha, China

**Keywords:** collective invasion and metastasis, epithelial ovarian cancer, LRRC4, PIK3R1, E-cadherin

## Abstract

Epithelial ovarian cancer (EOC) is the most malignant gynecological carcinoma and is of a high incidence of death due to detection at late stages when metastasis already occurs. However, the mechanism underlying metastasis of EOC remains unclear. Analysis of the open database and experiments with immunochemistry showed that LRRC4 is lowly expressed in high-grade serous ovarian cancer (HGSC) cells and during EOC metastasis. The 3D cell culture system and the orthotopic ovarian xenograft model infected with LRRC4-containing adeno-associated virus serotype 9 (AAV9) were used to confirm collective invasion and metastasis of cells *in vitro and in vivo*. Phos-tag SDS-PAGE was used to detect the phosphorylation of LRRC4 and PIK3R1. A number of experiments with methods such as co-immunoprecipitation and immunoblotting were performed to explore the mechanism for the actions of LRRC4 and PIK3R1 in EOC metastasis. An inverse correlation between LRRC4 and E-cadherin expression was detected in the regions of invasion in primary EOC tissues and metastatic ascites. LRRC4 binds to the cSH2 domain of PIK3R1 and inhibits the activity of PIK3R1, without disrupting the physical interactions between PIK3R1 and PIK3CA. LRRC4 inhibits EOC metastasis by targeting E-cadherin-dependent collective cell invasion and does so by inhibiting the PIK3R1-mediated AKT/GSK3β/β-catenin signaling pathway. LRRC4 functions as a tumor suppressor gene to inhibit EOC collective invasion and metastasis *in vitro* and *in vivo* and does so by directly binding to the cSH2 domain of PIK3R1 to exert its regulatory function. Our findings provide a potential novel approach for metastasis prognosis and a new strategy for the treatment of EOC.

## Introduction

Epithelial ovarian cancer (EOC) is the most malignant gynecological carcinoma with a high mortality rate and poor prognosis (1). An increasing trend in ovarian cancer mortality has been observed in females (1). High-grade serous ovarian cancer (HGSC) accounts for ~75% of all ovarian cancer deaths due to advanced stage and cancer cell metastasis when patients are diagnosed (2). In the past 30 years, little improvement in overall survival has be achieved, while the standard treatment has not advanced beyond platinum-base combination chemotherapy (3). Despite considerable efforts to improve early detection and treatment, tumor metastasis remains a major challenge for clinical treatment (4). Metastasis is a complex and multistep process that requires cancer cells to detach from primary tumors and migrate to distant organs and is responsible for more than 90% of all cancer-associated deaths. However, EOC mostly metastasizes into the peritoneal cavity as individual cells and clusters by shedding from ovarian cancer instead of spread in haematogenously (5, 6). Recent studies have revealed that cell-collective metastasis is the primary mechanism for metastasis in ovarian cancer and that cell-cell junction plays a key role in collective cell movement (7, 8).

More recently, genomic analyses have revealed that the alteration of the PI3K/AKT pathway in HGSC is a common event and is associated with poor clinical outcomes (9). The regulatory subunit, PIK3R1 (p85α), of PI3K tightly interacts with PIK3CA, the catalytic subunit of PI3K-p110α (as heterogeneity in nSH2 or cSH2 of PIK3R1), resulting in the inhibition of PI3K-p110α activity (10). Moreover, inhibition of PI3K-p110α is released when PI3K-p110α is recruited to the plasma membrane by receptor tyrosine kinases (RTKs), tyrosine-phosphorylated adaptor proteins, and the members of the Ras superfamily of small G proteins (11), leading to the activation of downstream effectors, such as AKT (12). PIK3R1 is currently considered as an oncogene in ovarian cancer (13).

LRRC4 (Leucine-rich repeat-containing protein 4) is a transmembrane and cytoplasmic protein and plays an important role in neural development and malignant transformation of glioma (14–18). We had previously shown that LRRC4 binds to PAR6 and serves as a partner of PAR complex in the neuron polarity (14). LRRC4 deletion contributes to glioblastoma multiforme (GBM) malignant progression, while LRRC4 overexpression inhibits GBM cell proliferation and invasion by inhibiting the RTK/ERK/AKT/NF-kB signaling pathway (15–18). LRRC4 binds to ERK1/2 directly and recruits ERK1/2 to the cytoplasm (17). In addition, we previously unveiled signaling loops involving LRRC4, AP-2, miR-182, and LRRC4 (the LRRC4-AP-2-miR-182-LRRC4 loop) and LRRC4, miR-185, SP1, DNMT1, and LRRC4 (the LRRC4-miR-185/SP1-DNMT1-LRRC4 loop), which play important role in glioma (18). Moreover, in addition to a role in severe intellectual disability and autism (19), it has been reported that LRRC4 is involved in nasopharyngeal carcinoma and pituitary adenoma (20, 21). LRRC4 has an established function in malignant transformation.

In this study, we examined the role of LRRC4 in EOC metastasis. We detected low LRRC4 expression in HGSCs and metastatic tissues. In ovarian cancer orthotopic xenograft, LRRC4 overexpression inhibits metastasis of ovarian cancer cells. The inhibition of metastasis is dependent upon E-cadherin, but independent of EMT. At the mechanistic level, we showed that LRRC4 binds to the c-SH2 domain of PIK3R1 to inhibit the activity of PIK3R1 and subsequently disrupts E-cadherin-dependent collective invasion of ovarian cancer cells mediated by the PIK3R1 signaling pathway.

## Materials and Methods

### Cell Culture and Tissue Samples

A2780 human ovarian cancer cells were obtained from BOSTER Biological Technology. SKOV3, OVCAR3, and OVCAR5 cells were obtained from the Cell Culture Center of PUME. HEK293, A2780, and HO8910 cell lines were cultured in high-glucose DMEM (Invitrogen, CA, USA) containing 10% fetal bovine serum at 37°C in 5% CO_2_. SKOV3, OVCAR3, and OVCAR5 cell lines were grown in RPMI medium (Invitrogen, CA, USA) containing 10% fetal bovine serum at 37°C in 5% CO_2_. Paraffin-embedded specimens of serous cystoma, HGSC and omental metastasis tissues were obtained from the Second Xiangya Hospital of Central South University and with appropriate patient consent.

### Vector, shRNAs, and siRNAs

The recombinant adeno-associated virus serotype 9 (AAV9) vector of LRRC4 (AAV9/GFP/LRRC4) was obtained from Weizhen biotechnology limited company. shRNA constructs against PIK3R1 and a non-targeting shRNA control were obtained from the BROAD Institute. Transient knockdown of LRRC4 in SKOV3 cells was performed using siRNAs purchased from GenePharma. FOP/TOP plasmids were obtained from Majian group of Cancer Research Institute in Central South University.

### Stable Cell Lines

The pcDNA3.1-LRRC4 plasmid was transient transfected into SKOV3 cells, and cells were thereafter treated with G418 for one weak in order to select the SKOV3 cells with pcDNA3.1-LRRC4 stable expression. In addition, lentivirus, which was generated by HANBIO and carried GFP or GFP/LRRC4, was used to infect the SKOV3 cells. Cells with stable expression of GFP or GFP/LRRC4 SKOV3 were selected by the use of flow cytometry.

### Immunohistochemistry Staining

All tissues were fixed in 4% paraformaldehyde and embedded in paraffin. For samples from human HGSC, metastasis and ascitic tissues, the anti-LRRC4 (Santa Cruz) and anti-E-cadherin (Cell Signaling Technology) primary antibodies were used at dilutions of 1:300 and 1:500, respectively. For mouse tissues, the anti-ki67 (Santa Cruz) primary antibody was used at 1:200. LRRC4 (Abcam), E-cadherin (Cell Signaling Technology), pAKT (ser473) (Cell Signaling Technology), AKT (Proteintech), GSK3β (Cell Signaling Technology), pGSKβ (ser9) (Cell Signaling Technology), Vimentin (Boster Biological Technology), and β-catenin (Sangon Biotech) primary antibodies were used 1:500. A biotinylated secondary antibody was used to recognized primary antibodies, and signals were visualized using the Ultra Sensitive TM SP system and peroxidase substrate DAB kit. Immunohistochemistry data were quantitated using staining intensity and proportion of tumor cells. The intensity was scored using a system from 0 to 3 as follows: negative as 0; weak as 1; moderate as 2; and strong as 3. The frequency of positive cells was defined using a system from score 1–4 as follows: negative or <5% as 0; 5–25% as 1; 26–50% as 2; 51–75% as 3; and >75% as 4 score. Each component was scored independently, and the results were summed up.

### Orthotopic Xenograft

Briefly, mice were completely anesthetized by the use of pentobarbital, and a dorsolateral incision (1–2 cm long) was made using surgical scissors on the top right of the spleen. The fat pad was exposed to open access to the mouse ovary. The ovary was firmly grasped with forceps, and 10^6^ tumor cells were injected into the ovary. After forceps were released, the wound was closed using sutures. After mice were sacrificed, the tumors were dissociated from the mouse ovary and omentum, while the size of the tumors was measured. For the intraperitoneal xenograft, 2 × 10^6^ tumor cells were intraperitoneally implanted into 5–6 week-old mice, which were euthanatized after 8 weeks. The metastasis nodules were quantified, while the number of mice used in each experiment is depicted in figure legends.

### Cell Proliferation and Invasion Assays

Proliferation was measured using EdU (EdU Apollo^®^488 *in vitro* Imaging Kit). Briefly, 5,000 cells/well were seeded in a 96-well plate in 100 μl of growth medium 24 h post-transfection. After 36 h, cell proliferation was measured according to the manufacturer's instructions. For the invasion assay, 10^5^ cells were placed in the top compartments medium containing 1% FBS while medium containing 10% FBS was placed in the bottom compartments of a Boyden chamber (Corning). After 36 h, cells that invaded through the membrane were stained with crystal violet and counted using Image J.

### Quantitative Real-Time PCR

Total RNA was extracted using the RNeasy Mini Kit (Qiagen). Total RNA (1 μg) was processed to generate cDNA using the Revert Aid First Strand cDNA Synthesis Kit (Thermo Fisher). Quantitative real-time PCR (RT-PCR) was conducted using a Bio-Rad CFX96 system with SYBR green (Takara) to determine the mRNA levels of genes of interest.

### Immunoblotting

For total cell lysates, cells were lysed in lysis buffer, and proteins were quantified using a BCA assay. For nuclear and membrane proteins, proteins were separated using a kit (Beyotime Biotechnology and Thermo). Total protein (30 μg) was separated on 8–10% SDS/PAGE gels and then transferred onto polyvinylidene fluoride PVDF membranes (Millipore, Billerica, MA). PVDF membranes were blocked with 3–5% BSA for 1 h and then incubated with specific primary antibodies at 4°C overnight. The primary antibodies used were the same as those used for immunohistochemistry. Membranes were incubated with HRP-conjugated secondary antibodies (Proteintech) and visualized using the ECL system (Millipore). Immunoblots were developed using a ChemicalDocTM XRS+ (Bio-Rad, Berkeley, CA, USA).

### Co-immunoprecipitation

Cells were lysed with immunoprecipitation buffer containing protease inhibitors. Lysates were incubated with 6 μg/ml antibodies or normal IgG at 4°C overnight in a rotary agitator. Protein A magnetic beads were added to lysates and incubated for 4–6 h at 4°C. Magnetic beads were collected and washed three times with immunoprecipitation buffer. Total lysates and immunoprecipitants were separated by SDS/PAGE gel and analyzed using western blotting.

### Immunofluorescence

Cells were fixed in 4% paraformaldehyde for 20 min at room temperature, permeabilized with 0.25% Triton-X, and blocked in 5% bovine serum albumin (BSA) for 30 min. Cells were incubated with primary antibodies at 4°C overnight or 6–8 h at room temperature. Secondary antibodies were coupled with Alexa Fluor 488 and 647, and cells were incubated with secondary antibodies for 2 h at room temperature. Cells were also incubated with phalloidin at 1:1,000 to stain F-actin-containing cells.

### 3D Cell Culture

Cells were digested into single-cell suspensions at a density of 3,000 cells/ml in conditioned medium. Cells were then embedded in Matrigel (BD Biosciences, 354236) or Collagen I (BD Biosciences, 354236) with 100 ng/ml EGF, 20 ng/ml FGF, 2% B27, and 1% antibiotics (100 units/ml penicillin and 100 mg/ml streptomycin) in 200 μl of medium. Cells were then placed in a 37°C heating block for 3 days, and the medium mixture was replaced every 2 days.

### Phos-tag SDS-PAGE

Cells were lysed in phosphorylation buffer, and protein was quantified. The SDS-PAGE system used to detect the phosphorylation of LRRC4 and PIK3R1 consisted of a separating gel and a stacking gel. The separating gel (7.5 mL) consisted of 6%w/v polyacrylamide and 1.5 mM Bis-Tris–HCl buffer (pH 8.8) and was mixed with 30 μM Phos-tag and two equivalents of MnCl2. The stacking gel (2.5 mL) consisted of 4.5% w/v polyacrylamide and 1.4 mM Bis-Tris–HCl buffer (pH 6.8). Western blotting was performed, and primary antibodies were incubated with the membranes.

### Statistical Analysis

Student's *t*-test was used to assess the significance of mean differences using SPSS Statistics software. Differences were considered significant at a two-tailed *P*-value of 0.05. All experiments were performed at least three times.

## Results

### Low LRRC4 Expression Is Associated With HGSC and EOC Metastasis

**Figure 1:**
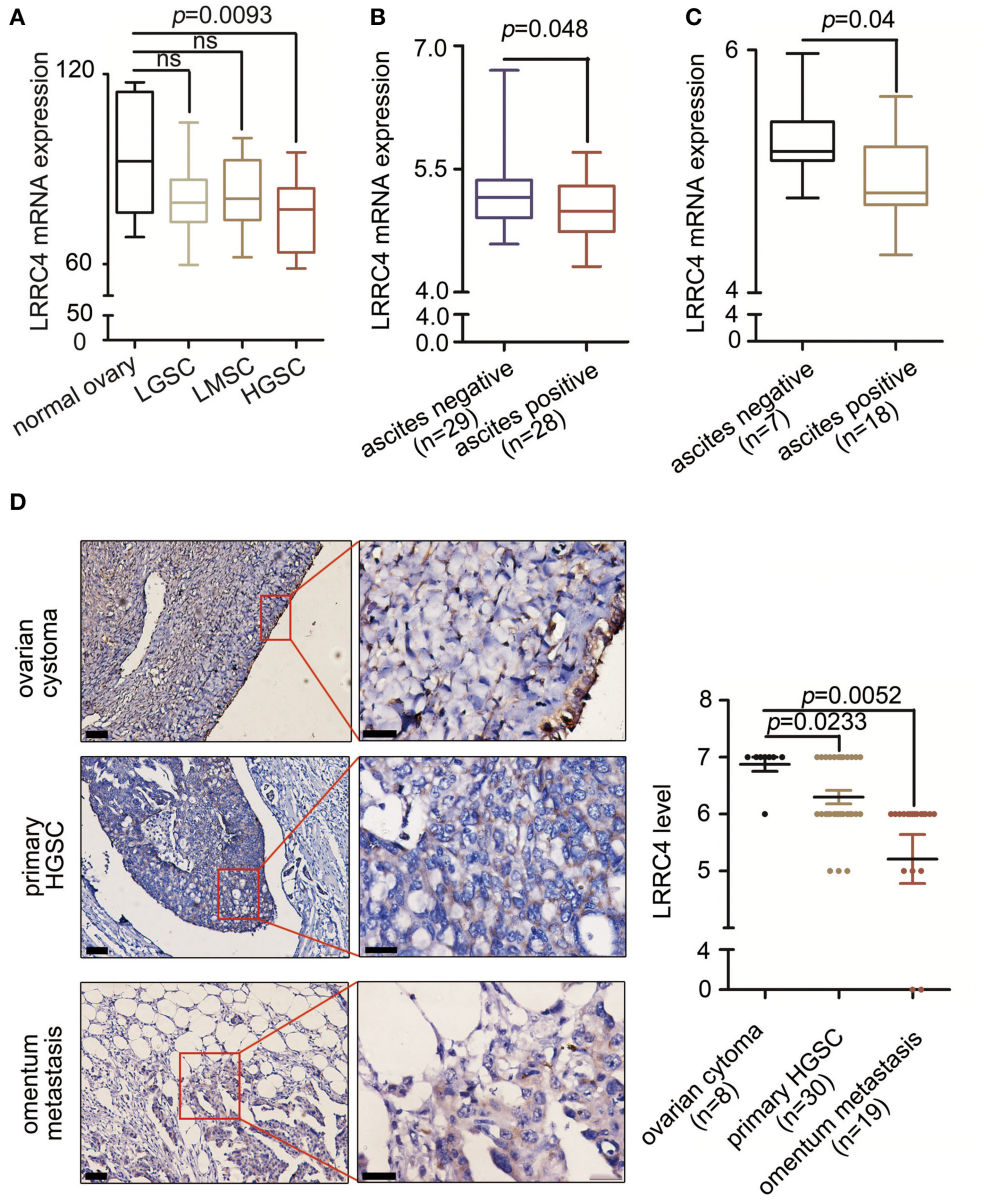
Low LRRC4 expression is associated with HGSC and EOC metastasis. **(A)** LRRC4 expression in human serous ovarian cancer (GSE92780) correlated inversely with high serous ovarian cancer. Samples include those from normal ovary (*n* = 5), low grade serous ovarian cancer (LGSC) (*n* = 11), low malignant serous ovarian cancer (LMSC) (*n* = 8), and high grade serous ovarian cancer (HGSC) (*n* = 17). **(B,C)** Low LRRC4 expression is associated with positive ascitic cytology in epithelial ovarian cancer (EOC) or serous ovarian cancer, based on the analysis of GSE39204 dataset. **(D)** Low expression of LRRC4 in high serous ovarian cancer (*p* = 0.0233) and omentum metastasis tissue (*p* = 0.0052) compared with that in ovarian cystoma tissues. Representative IHC images of LRRC4 staining in ovarian cystoma (*n* = 8), primary HGSC (*n* = 30) and omentum metastasis tissues (*n* = 19). Scale bars: the left is 50 μm while the right is 20 μm. The area in red boxes to the right is magnified.

We first examined the expression of LRRC4 in 5 normal ovarian tissues, 11 low-grade serous ovarian cancer (LGSC), 8 low malignant serous ovarian cancer (LMSC), and 17 HGSC samples using Gene Expression Omnibus (GEO) databases. Compared with normal ovarian surface epithelial cells, LRRC4 expression was reduced in advanced HGSC (*p* = 0.0093), but no significant difference in LRRC4 expression was found between the normal ovarian surface epithelial cells and LGSC or LMSC cells (Figure 1A). LRRC4 was down-regulated in EOC tissues from ascitic cytology-positive patients compared with those from ascitic cytology-negative patients (Figure 1B). LRRC4 expression was also reduced in serous ovarian cancer cells with ascitic cytology-positive patients (Figure 1C), suggesting that low expression of LRRC4 is associated with high-degree EOC malignant progression and metastasis.

We next examined LRRC4 expression in 8 serous cystoma, 30 primary HGSC, and 19 omental metastasis samples by using immunochemistry (IHC), verifying that LRRC4 was significantly down-regulated in primary HGSC (*p* < 0.05) and omental metastasis samples (*p* < 0.01) compared with serous cystoma samples. There was a statistically significant difference between primary ovarian cancer and omental metastasis samples (*p* < 0.001) (Figure 1D).

### LRRC4 Suppresses EOC Tumourigenesis and Metastasis

**Figure 2:**
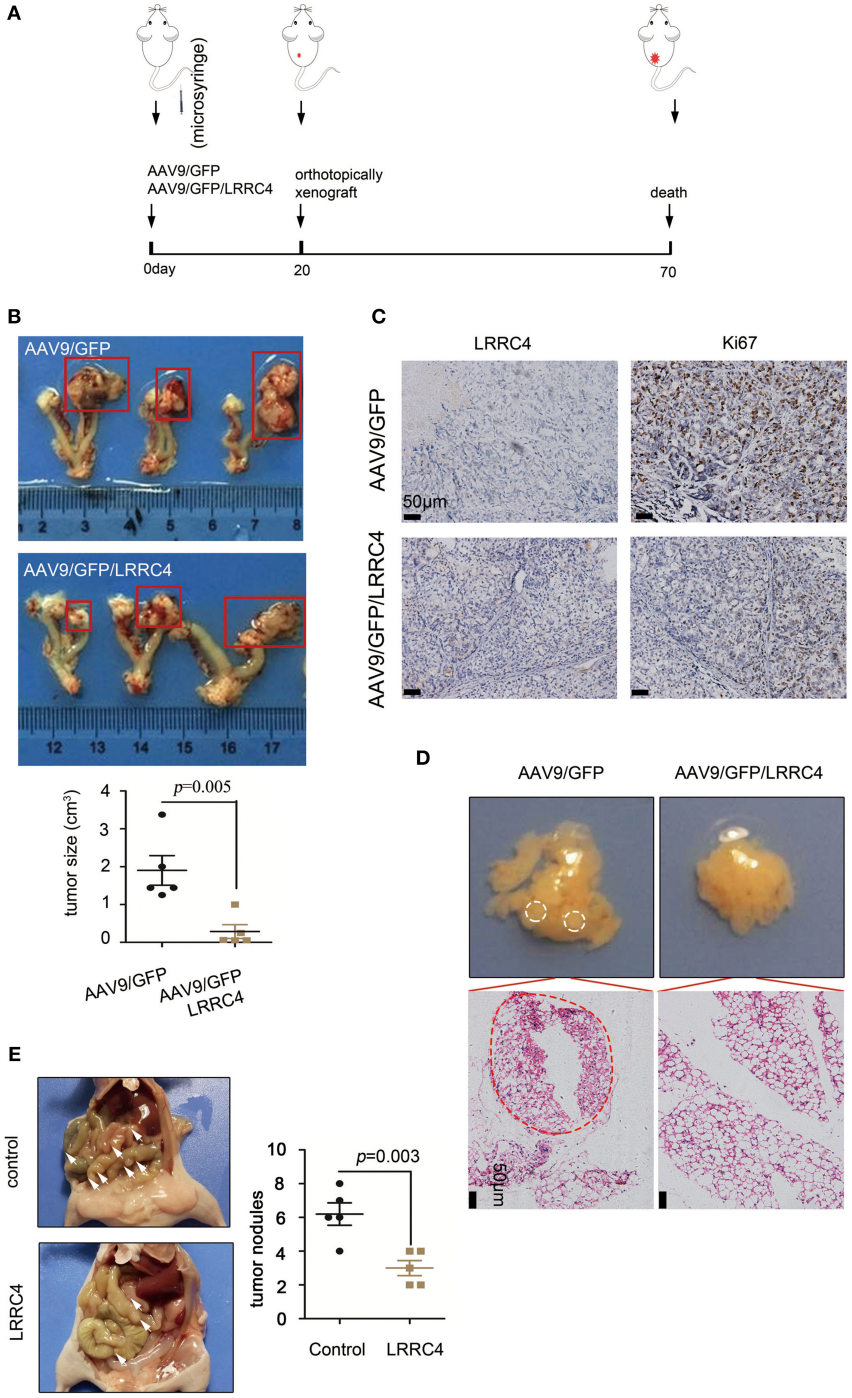
LRRC4 suppresses EOC tumourigenesis and metastasis. **(A)** Schematic representation of the mouse model. Mice were treated with adeno-associated virus for control (AAV9/GFP) or LRRC4 (AAV9/GFP/LRRC4) (*n* = 5 per group) for 20 d, and then mice were orthotopically xenografted with 106 SKOV3 EOC cells per mouse in each group. After 7 weeks, mice were analyzed. **(B)** Representative images showing tumor growth and tumor volumes. **(C)** Ki67 staining was performed in orthotopically xenografted primary tumors in mice. Scale bars: 50 μm. **(D)** Representative images showing omentum metastasis of cancer cells. The omentum was stained with haematoxylin and eosin (HE), and the results showed that LRRC4 inhibits ovarian cancer cell metastasis. Scale bars: 50 μm. **(E)** The intraperitoneal xenograft mouse model was generated using 2 × 106 SKOV3 cells transduced with empty vector or SKOV3 cells stably expressing LRRC4 (*n* = 5 per group). Vector-transduced SKOV3 cells formed significantly larger abdominal metastases (as the arrows) compared with SKOV3 cells stably overexpressing LRRC4.

The correlation between LRRC4 low expression and high degree of EOC invasion and metastasis led us to ask whether LRRC4 has a function in these responses. Experiments with Western blotting and real-time quantitative PCR showed that LRRC4 expression was either absent or at low levels in five EOC cell lines (Figures S1A,C). We used the SKOV3 cell line to stably express LRRC4 was generated using the lentivirus-based system, which allowed LRRC4 to effectively express (Figure S1B). EDU and transwell assays showed that LRRC4 ectopic expression inhibited cell proliferation and invasion (Figures S1D,E). We next used the ovarian orthotopic xenograft mouse model to assess the effect of LRRC4 ectopic expression on EOC tumourigenesis *in vivo* (22). We inserted LRRC4 cDNA into a recombinant AAV9 vector, which produces high and sustained serum levels of transgene when delivered as in a single injection (22). Mice were treated with AAV9/GFP/LRRC4 fusion vector (*n* = 5) or the control AAV9/GFP vector (*n* = 5) by the use of intraperitoneal injection (23), while the left ovary was orthotopically xenografted with 10^6^ SKOV3 EOC cells in each experimental group (Figure 2A). We confirmed LRRC4 expression in the mouse ovary, fallopian tube muscle layer, fallopian tube mucosa, and xenografted tumors by using IHC, indicating that AAV9/GFP/LRRC4 was successfully expressed (Figure S2A). As shown in Figure 2B, the SKOV3 cells implanted orthotopically in the ovary formed tumors in the AAV9/GFP group, while the AAV9/GFP/LRRC4 group exhibited significantly reduced tumourigenesis. Much weaker nuclear staining of ki67 was observed in cells of the AAV9/GFP/LRRC4 group compared with those of the AAV9/GFP group (Figure 2C). Moreover, LRRC4 ectopic expression reduced cancer cell metastasis to the omentum from ovarian orthotopicallyxenografts (Figure 2D). Finally, we used an intraperitoneal xenograft mouse model, which showed that LRRC4 ectopic expression significantly reduced abdominal metastases of SKOV3 cells when compared with those with empty vectors (Figure 2E).

### LRRC4 Inhibits Collective Cells Invasion by Down-Regulating E-Cadherin Without EMT Induction

**Figure 3:**
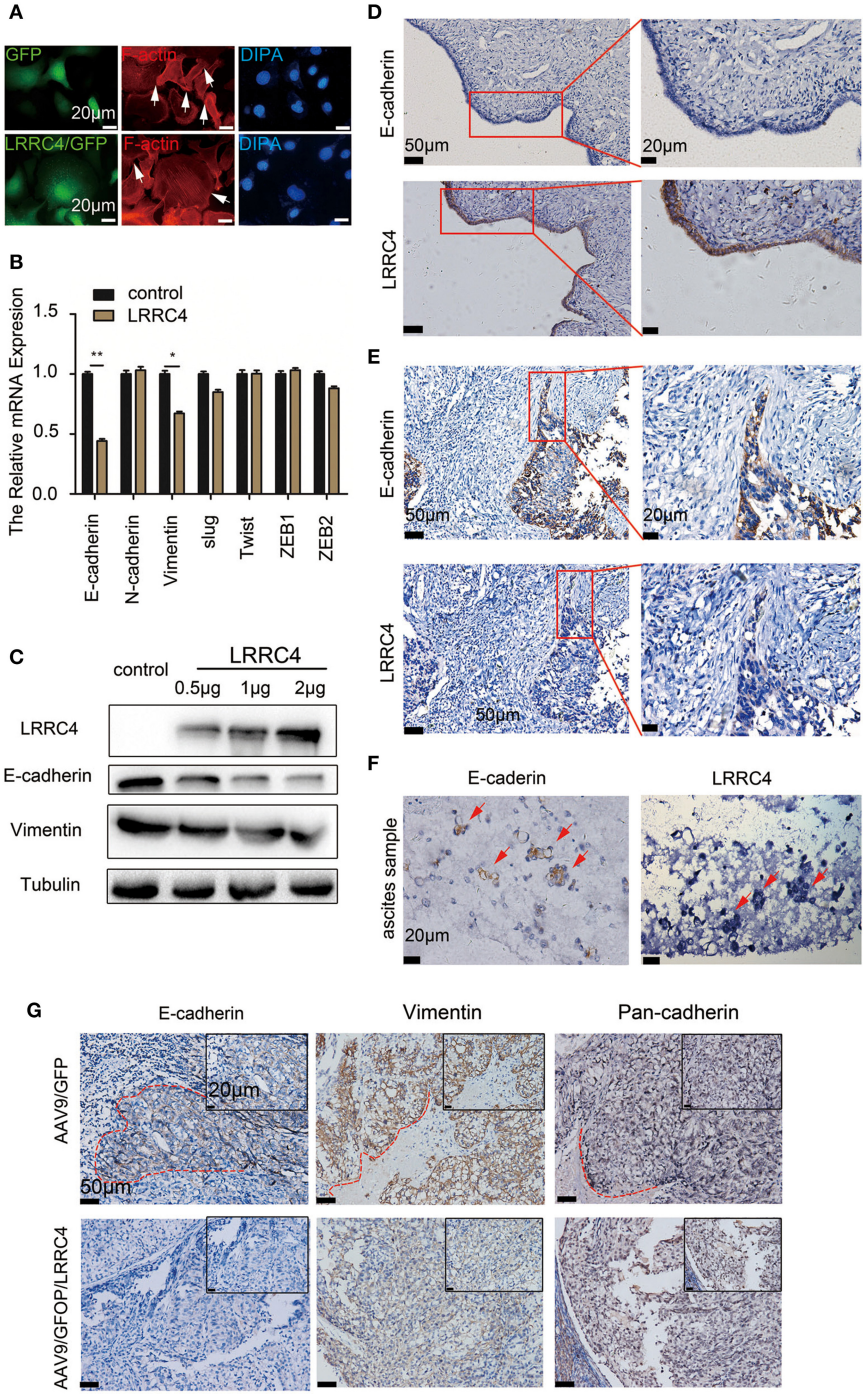
LRRC4 inhibits collective cells invasion by down-regulating E-cadherin without EMT induction. **(A)** Phalloidin staining was performed in SKOV3 cells stably expressing LRRC4 through lentivirus infection. F-actin aggregates along control cell peripherals, while LRRC4 inhibites the accumulation and aggregation of F-actin (indicated with arrowheads). **(B)** The mRNA levels of EMT associated-genes, including E-cadherin, N-cadherin, slug, twist, ZEB1 and ZEB2, as assessed with RT-qPCR. **(C)** Western blotting analysis of E-cadherin and Vimentin protein in SKOV3 cells transiently transfected with different doses of LRRC4 expression vector (0.5, 1, and 2 μg). **(D,E)** E-cadherin and LRRC4 protein expression and localization as detected with immunohistochemistry (IHC) in human normal ovarian epithelial cells (**D**, *n* = 5), human HGSC (**E**, *n* = 17) and ascites of ovarian cancer patients. Scale bars: the left is 50 μm while the right is 20 μm. The area in the red boxes to the right is magnified. **(F)** E-cadherin and LRRC4 in EOC ascitic tissue as stained with IHC. Scale bars: 20 μm. **(G)** Expression of E-cadherin, Vimentin and Pan-cadherin in intraperitoneal xenografted primary tumor tissues from the mouse model was examined by the use of IHC in. Scale bars: 20 μm and 50 μm. ^*^p < 0.05, ^**^p < 0.01.

How does LRRC4 inhibit EOC metastasis? Epithelial-mesenchymal transition (EMT), in which cells typically lose cell-cell cadherin and gain migration and invasion ability, plays a crucial role in tumor metastasis (24). The central role of EMT in tumor metastasis led us to assess whether it also mediates LRRC4 inhibition of EOC metastasis. Experiments with phalloidin staining demonstrated that SKOV3 EOC cells highly polarized with F-actin accumulation, while LRRC4 ectopic expression inhibited F-actin accumulation (indicated by arrowhead) compared with control cells (Figure 3A). At the molecular level, ectopic expression of LRRC4 simultaneously reduced the mRNA levels of E-cadherin and Vimentin but did not affect those of N-cadherin, slug, twist, ZEB1, and ZEB2 (Figure 3B). Western blotting also showed that LRRC4 ectopic expression reduced E-cadherin and Vimentin protein levels in a dose-dependent manner (Figure 3C). The failure of LRRC4 expression to up-regulate the EMT markers suggested that LRRC4 inhibits EOC cell metastasis independently of EMT.

If EMT does not mediate LRRC4 inhibition of EOC metastasis, what may be the mechanism? We found that human normal ovary epithelial cells exhibited relatively low E-cadherin but high LRRC4 expression (*n* = 5, Figure 3D). In contrast, EOC cells exhibited high E-cadherin but low LRRC4 expression in the invasion regions in human HGSC (*n* = 17, Figure 3E) and were organized into multicellular units, indicating collective invasion, and metastasis (red box). Furthermore, E-cadherin expression was present in the ascites of ovarian cancer patients and was accompanied by low LRRC4 expression (Figure 3F). Finally, we examined E-cadherin expression in EOC orthotopic xenograft tumor models we previously generated using AAV9/GFP. We detected high E-cadherin expression in tumor epithelial cells undergoing collective invasive (Figure 3G; red circle), but low E-cadherin and Vimentin expression in EOC orthotopic xenograft tumor models with AAV9/GFP/LRRC4. Together, these results showed that LRRC4 significantly reduces E-cadherin and Vimentin expression and collective invasion of EOC (Figure 3G) and that the expression of pan-cadherin and N-cadherin in the invasive cells (Figure 3G, column 3, red circle) (Figure 3B) remained unaffected. Thus, LRRC4 inhibits collective cell invasion by targeting E-cadherin.

### LRRC4 Inhibition of EOC Invasion Is Mediated by the PIK3R1/AKT/GSK-3β/β- Catenin Signaling Pathway

**Figure 4:**
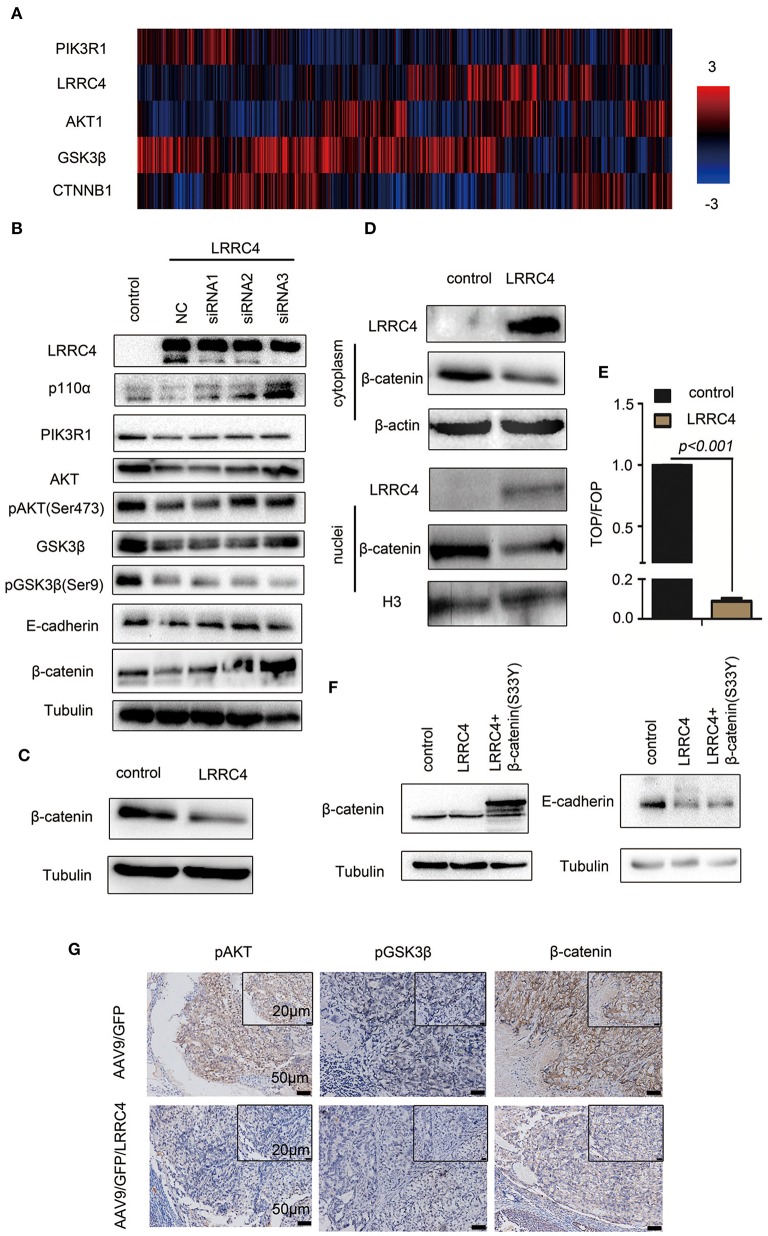
LRRC4 inhibition of EOC invasion is mediated by the PIK3R1/AKT/GSK-3β/β-catenin signaling pathway. **(A)** Expression of LRRC4 in human ovarian cancer was analyzed by the use of cBioportal of the TCGA database. LRRC4 expression correlated negatively with PIK3R1 (p85α), AKT1, GSK3β, and CTNNB1 (β-catenin) expression. **(B)** LRRC4-siRNAs were transfected into SKOV3 cells containing the control vector or LRRC4, and the cell lysates were immunoblotted for indicated proteins. **(C)** Whole-cell lysates were prepared, and β-catenin protein was analyzed with immunoblotting. **(D)** Cytoplasmic and nuclear proteins were separated by the use of a nuclear and cytoplasmic protein extraction kit and immunoblotted with indicated antibodies. **(E)** HEK293 cells overexpressing LRRC4 or containing the control vector were transfected with TCF/LEF and FOP/flash or TOP/flash. FOP/flash was a negative control. The ratio of TOP/FOP is shown in the chart. **(F)** Transient transfection of constitutively active β-catenin (S33Y) in SKOV3 cells stably expressing LRRC4 and immunoblotted with indicated antibodies. **(G)** IHC assay showing the expression of pAKT, pGSK3β, and β-catenin in intraperitoneal xenografted primary tumor tissues from the mouse model. Scale bars: the bottom is 50 μm while the top is 20 μm.

We next investigated the potential mechanism by which LRRC4 regulates E-cadherin-dependent collective invasion. The PI3K/AKT pathway is one of the most frequently altered pathways in EOC (25), leading us to assess the link between LRRC and the PI3K pathway. We first conducted genomic analyses of 489 human ovarian serous cystadenocarcinomas using the Cancer Genomics Atlas (TCGA), which revealed an inverse relationship between LRRC4 and PIK3R1 expression (Figure 4A). The inverse relationship between LRRC4 and PIK3R1 led us to ask whether LRRC4 mediates other components of the PI3K/AKT signaling pathway. Indeed, the expression of LRRC4 also inversely correlates with that of AKT1, GSK3β, and CTNNB1 (β-catenin) (Figure 4A).

We next examined the effects of LRRC4 overexpression/depletion on the components of the PI3K/AKT pathway. Overexpression of LRRC4 in SKOV3 cells (NC+pcDNA3.1-LRRC4, line2) significantly reduced the levels of p110α, PIK3R, AKT, pAKT (ser 473), GSK3β, pGSK3β (ser9), E-cadherin, and β-catenin compared with control cells without overexpression (pcDNA3.1 vector, line1) (Figure 4B). In contrast, knockdown of LRRC4 via small interfering RNAs (siRNA) in SKOV3 cells stably expressing LRRC4 rescued the down-regulation of p110α, PIK3R1, pAKT (ser473), AKT, GSK3β, E-cadherin, and β-catenin (Figure 4B, pcDNA3.1-LRRC4+ LRRC4 siRNA1, 2 or 3, lines 3,4 and 5), with LRRC4 siRNA3 showing the strongest knockdown efficacy. Interestingly, LRRC4 knockdown did not rescue the down-regulation of pGSK3β (ser9), implying that LRRC4 inhibits pGSK3β (ser9) through signaling pathways other than PI3K/AKT (Figure 4B, pcDNA3.1-LRRC4+ LRRC4 siRNA1, 2 or 3). It has been recently shown that cell adhesion molecules such as E-cadherin and P-cadherin play an important role in collective metastasis (26). In the adhesion complexes, β-catenin tightly binds to cadherin and is critical for cellular structures and actin cytoskeleton organization (27). During carcinogenesis and EMT, β-catenin translocates to the nuclei, enabling β-catenin to function as a transcriptional co-regulator and cooperate with transcription factors of the T-cell factor (TCF) family to repress E-cadherin expression and support EMT progress (28). We next focused on β-catenin and found that LRRC4 overexpression reduced β-catenin expression (Figure 4C) and prevented its nuclear translocation (Figure 4D). Experiments with the Top/Fop assay also demonstrated that LRRC4 overexpression markedly inhibited β-catenin activities (Figure 4E). However, when the β-catenin mutant (S33Y), which is refractory to phosphorylation and is therefore unable to be degraded by the proteasome, was expressed in SKOV3 cells, the down-regulation of E-cadherin caused by LRRC4 overexpression was rescued (Figure 4F), suggesting that LRRC4 controls E-cadherin expression through β-catenin. Consistent with the observations from *in vitro* experiments, the levels of pAKT, pGSK3β and β-catenin were lower in the orthotopic primary implantation sites of mice in the AAV9/GFP/LRC4 group than those in the AAV9/GFP group (Figure 4G).

### LRRC4 Binds to PI3KR1 and Inhibits PI3KR1 Phosphorylation

**Figure 5:**
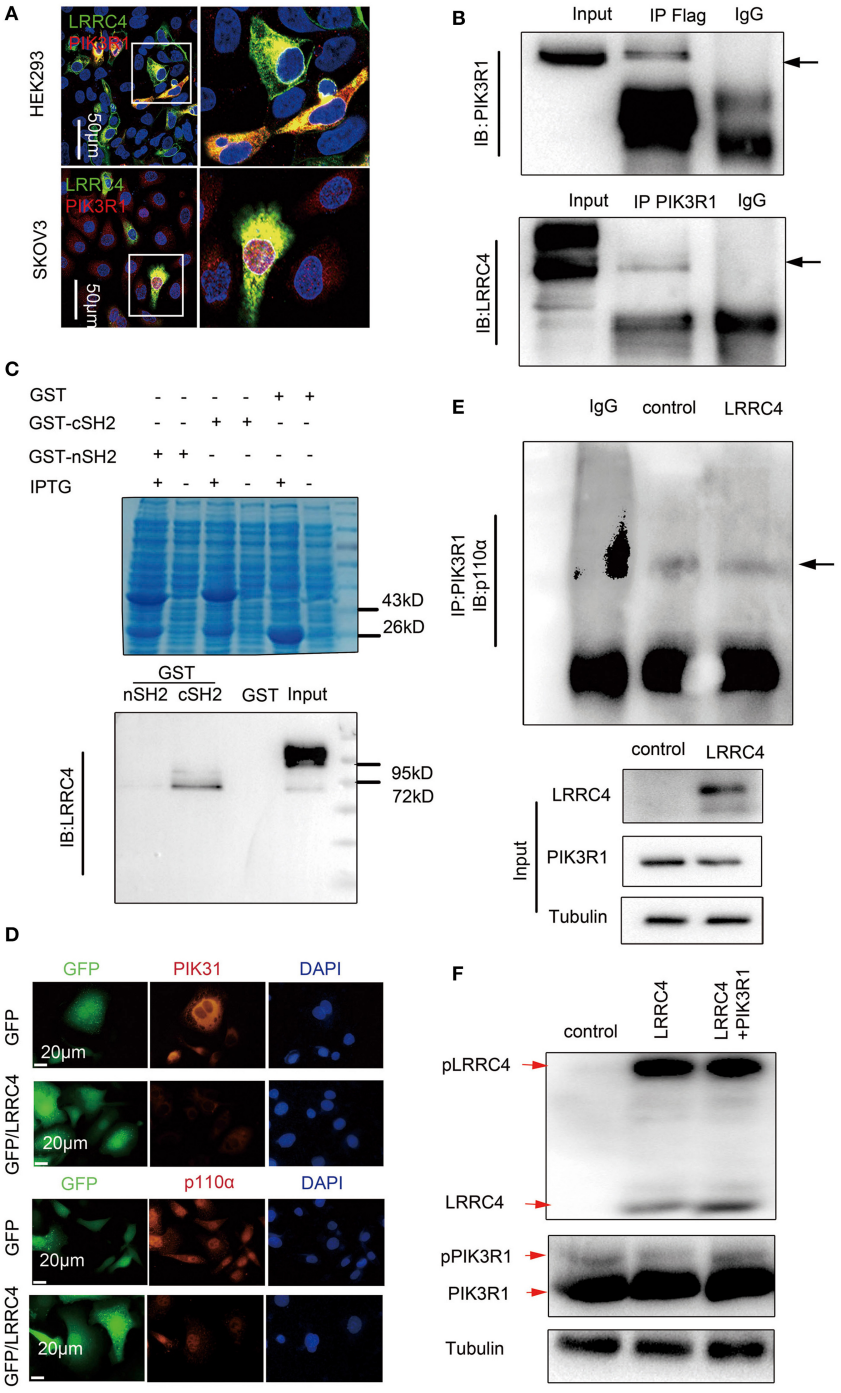
LRRC4 binds to PI3KR1 and inhibits PI3KR1 phosphorylation. **(A)** Immunofluorescence of LRRC4 and PIK3R1 in SKOV3 cells (top) and HEK293 cells (bottom). Confocal images showing the colocalization of LRRC4 and PIK3R1. Scale bars: 50 μm. **(B)** HEK293 cells were co-transfected with Flag-LRRC4 and HA-PIK3R1. Proteins were precipitated with human antibodies specific to control IgG, Flag, and PIK3R1 followed by immunoblotting analysis. **(C)** GST pull-down assays showing that LRRC4 directly binds to the cSH2 domain of PIK3R1. Coomassie blue staining of whole-cell lysate (top); Western blotting analysis of the expression of the GST fusion protein (bottom). **(D)** Staining of SKOV3 cells stably expressing LRRC4 or containing the control vector showing that LRRC4 reduces the levels of p110α and PIK3R1 proteins. Scale bars: 20 μm. **(E)** Cell lysates were prepared from SKOV3 cells stably expressing LRRC4 or containing the control vector, and qualitative analysis of immunoprecipitation showed that LRRC4 does not affect the interaction between PIK3R1 and p110α. **(F)** Cells were lysed and subjected to Phos-tag western blotting with LRRC4 and PIK3R1 antibodies.

How does LRRC4 regulate PI3K? We analyzed potential LRRC4 protein interactions using Scansite3 software, which predicted that PIK3R1 can putatively interact with LRRC4 and does so via the PIK3R1-SH2 domain (http://scansite3.mit.edu/). Experiments with immunofluorescence analysis showed that LRRC4 and PIK3R1 proteins colocalized in the plasma membranes and the cytoplasm in HEK293 cells, while both were only present in the cytoplasm of SKOV3 EOC cells (Figure 5A). We also found that LRRC4 co-immuoprecipitated with PIK3R1 in HEK293 cells (Figure 5B). Furthermore, we preformed the GST pull-down assay, which revealed direct binding between LRRC4 and PIK3R1 through the c-SH2 domain of PIK3R1 (Figure 5C).

PIK3R1 serves as the regulatory subunit of PI3K and complexes with PIK3CA (p110α), leading to the inhibition of PIK3CA activity and subsequently PI3K/AKT signaling, while the inhibition is prevented when RKTs recruit PI3K to the plasma membranes (16). Although LRRC4 functioned to suppress PIK3R1 and PIK3CA expression in SKOV3 cells, as shown by Western blotting (Figure 4B) and immunofluorescence analyses (Figure 5D), LRRC4 overexpression did not disrupt the interaction between PIK3R1 and PIK3CA (Figure 5E). It was previously shown that that PIK3R1 phosphorylation leads to increased membrane binding of PI3K and promotes PI3K activity when PIK3R1 complexes with the p110 subunit (29), leading us to assess the effect of LRRC4 onPIK3R1 phosphorylation. We detected phosphorylation of both LRRC4 and PIK3R1 using the Phos-tag SDS-PAGE analysis, which detects phosphorylated proteins based on differences in protein migration speed. PIK3R1 overexpression did not affect the level of LRRC4 phosphorylation, while LRRC4 overexpression reduced the level of PIK3R1 phosphorylation. The reduction in PIK3R1 phosphorylation was alleviated when PIK3R1 was ectopically expressed (Figure 5F, shown as red arrows).

**Figure 6:**
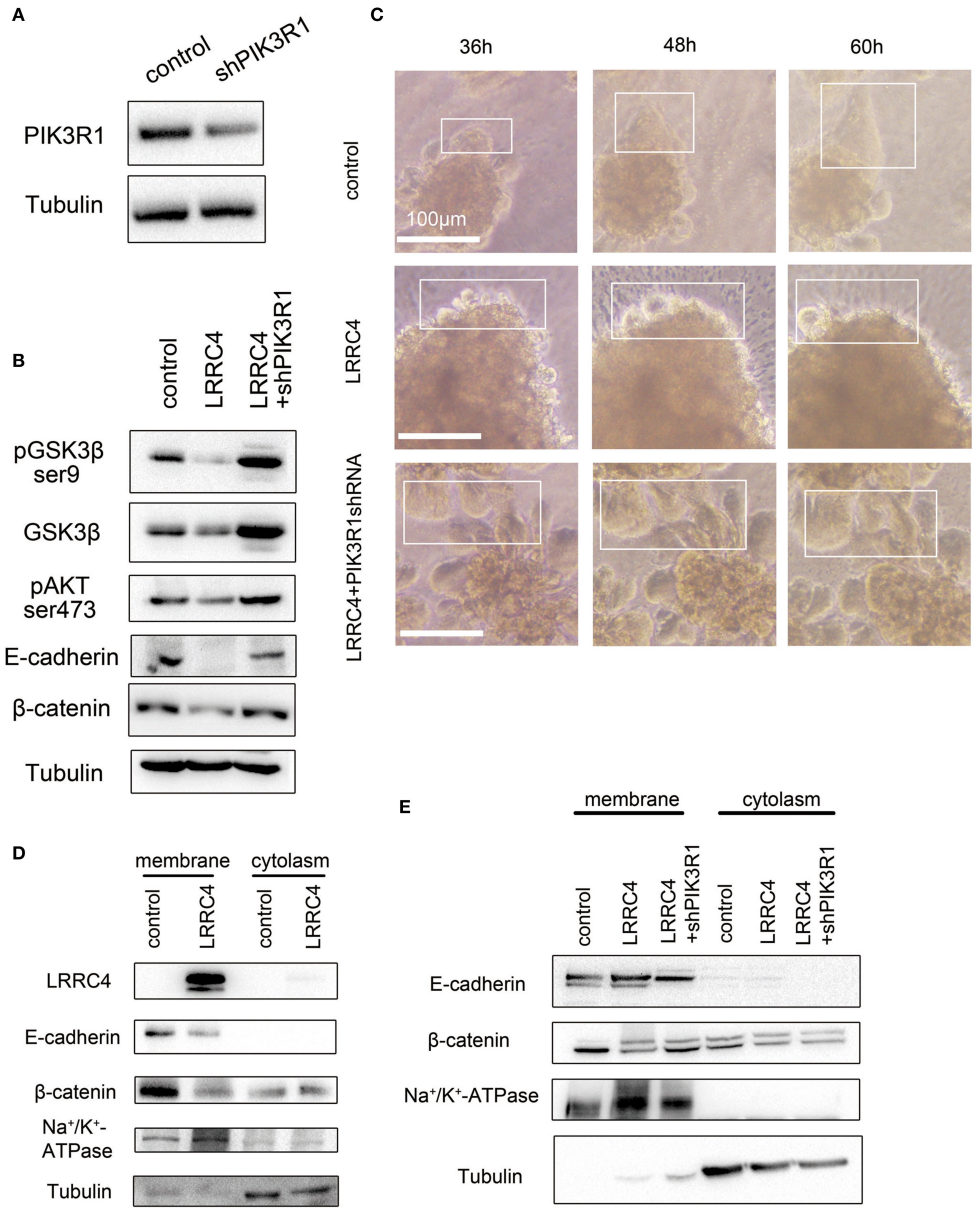
LRRC4 inhibits collective cell invasion via the PIK3R1 signaling pathway. **(A)** Western blotting analysis showing the efficiency of shPIK3R1 knockdown. **(B)** shPIK3R1 was transfected into SKOV3 cells stably expressing LRRC4, and lysates were subjected to immunoblotting analysis of indicated proteins. **(C)** PIK3R1 was knocked down in SKOV3 cells stably expressing LRRC4 or containing the control vector, and cells were then embedded in Matrigel (top) or Collagen I (bottom) to form 3D cell culture. Cells were allowed to invade for 36–60 h. Scale bars: 100 μm. **(D)** Membrane-bound and cytoplasmic proteins in control or LRRC4 expressing SKOV3 cells were separated by the use of a membrane and cytoplasmic protein extraction kit and immunoblotted with LRRC4, E-cadherin and β-catenin antibodies. N+K+-ATPase is a membrane protein marker, and tubulin is a cytoplasmic protein marker. **(E)** shPIK3R1 was transfected into SKOV3 cells stably expressing LRRC4, and membrane-bound and cytoplasmic proteins were separated and immunoblotted with LRRC4, E-cadherin and β-catenin antibodies.

To ask if LRRC4 suppression of pAKT, pGSK-3β, β-catenin, and E-cadherin functioned through PIK3R1, we generated a shRNA vector to knockdown PIK3R1 in SKOV3 cells (Figure 6A). When PIK3R1 was depleted, the suppressive effects of LRRC4 overexpression on pGSK-3β, pAKT, β-catenin, and E-cadherin expression were rescued compared with LRRC4 overexpression alone (NC+pcDNA3.1-LRRC4 line 2) (Figure 6B). Experiments with the 3D culture system also showed that LRRC4 suppression of cell collective movement was alleviated 36–60 h after cultivation culture with PIK3R1 knockdown (Figure 6C, shown as the box).

The E-cadherin-Catenin adhesion Complex can be found in the plasma membranes and in the cytoplasm. To ask which pool of E-Cadherin and β-catenin was reduced after LRRC4 overexpression, we separated plasma membranes and cytoplasm and determined the levels of the two proteins. We found that LRRC4 overexpression caused the levels of membrane-associated E-cadherin and β-catenin to significantly reduce while exerting little on the cytoplasmic pool of the two proteins (Figure 6D). As expected, the reduction in E-cadherin and β-catenin levels in the plasma membranes caused by LRRC4 overexpression was rescued after knockdown of PIK3R1 (Figure 6E). Together, these results suggested that LRRC4 inhibits collective invasion and metastasis of EOC by targeting membrane-bound E-cadherin via the PIK3R1/AKT signaling pathway.

## Discussion

The high mortality of EOC is mainly due to detection at late stages with vast intraperitoneal dissemination and to chemotherapy resistance (30). Ovarian cancer often metastasizes to the omentum, intestine, and liver through the peritoneal cavity with peritoneal fluid dissemination (31), in which cancer cells directly extend from primary tumors and metastasize to peritoneal tissues as a single cell or clusters (4). Nevertheless, haematogenous metastasis also exists with circulating tumor cells (CTCs) found in ovarian cancer patients (6, 32). Epithelial ovarian cancer metastasis occurs prior to a predominantly intraperitoneal dissemination mechanism (4, 33). Here we present evidence to show that decreased LRRC4 expression modulates EOC collective invasion and metastasis, especially to omentum, and ascites. Thus, LRRC4 functions as a tumor suppressor gene that inhibits EOC tumourigenesis and omentum metastasis.

Cancer cell metastasis is a complex process and involves various molecular mechanisms (34). The mechanism for cancer cell metastasis can be grouped into two categories including single cell migration and collective cell migration, in which cells move individually or as clusters, respectively (35). Previous studies have indicated that collective cell metastasis occurs through cell connections via cell-cell junctions, mostly cadherin-mediated cell-cell interactions, to enable coordinated cell movement by following the leader cells (36). Several studies have suggested that E-cadherin or P-cadherin plays important roles in junctions during collective metastasis (37). In addition, in-depth single-cell phenotypic characterization of high-grade serious ovarian cancer has shown that cancer cells co-express E-cadherin and vimentin, which correlates significantly with tumor recurrence and metastasis (38). Although EMT and MET are methods of tumor metastasis in ovarian cancer patients (39), our current study shows that LRRC4 acts as a suppressor gene to inhibit EOC cell collective invasion in an E-cadherin-dependent manner but not through EMT. This is mainly because LRRC4 ectopic expression not only simultaneously inhibits the expression of E-cadherin and Vimentin *in vitro* and *in vivo* but also attenuates EOC collective invasion and omentum metastasis. In primary EOC tissues, cell collective invasion exists, while high-level E-cadherin expression and low-level LRRC4 expression are present in the region of collective invasion. In addition, high-level expression of E-cadherin is also accompanied by the loss of LRRC4 expression in cancer cell clusters in ascites of EOC patient.

PI3K transduces phosphatidylinositol-4,5-bisphosphate (PI-4,5-P2) to generate phosphatidylinositol-3,4,5-trisphosphate (PIP3) and then actives AKT kinase (40). The PI3K/AKT pathway is frequently altered in human cancer and is critical in tumor initiation and progression (41). As the regulatory subunit of PI3K, PIK3R1 maintains PI3K in a low activity state via interacting with the PIK3CA catalytic subunit (42). GSK3β allows crosstalk between PI3K/AKT and Wnt pathways and antagonizes Wnt signaling by forming protein complexes with Axin1 and CK1 to polyubiquitinate β-catenin, the central component of canonical Wnt signaling, to inhibit cancer cell proliferation and invasion (43, 44). Our study shows that LRRC4 binds to the cSH2 domain of PIK3R1 and inhibits the expression of PIK3R1 and PIK3CA and that LRRC4 does not affect the interaction between PIK3R1 and PIK3CA. LRRC4 does not only repress the phosphorylation of PIK3R1 but also inhibits pAKT, pGSK3β, and β-catenin expression and subsequently collective cell invasion. Moreover, during EMT, β-catenin translocates from the cytosol to the nuclei, allowing it to function as a transcriptional co-regulator and cooperate with transcription factors of the TCF family to repress E-cadherin expression and support EMT (28). Although LRRC4 represses β-catenin expression and nuclear translocation, LRRC4 mainly attenuates E-cadherin and β-catenin levels in the plasma membranes. Because β-catenin binds tightly to the cytoplasmic domain of type I cadherins, such as E-cadherin, and plays an essential role in the actin cytoskeletal organization (27), the LRRC4/PIK3R1 axis may inhibit the intrinsic invasive ability of ovarian cancer cells to and reduce tumor metastasis behavior by disrupting cell-cell junctions via the AKT/GSK-3β/β-catenin/E-cadherin signaling mechanism.

## Conclusions

In summary, we find that LRRC4 is significantly down-regulated and is closely associated with metastasis in EOC patients. LRRC4 functions as a tumor suppressor gene to inhibit EOC collective invasion and metastasis *in vitro* and *in vivo* and does so by directly binding to the cSH2 domain of PIK3R1 to exert its regulatory function. Our findings provide a potential novel approach for metastasis prognosis and a new strategy for the treatment of EOC.

## Data Availability Statement

Publicly available datasets were analyzed in this study. This data can be found here: http://scansite3.mit.edu/.

## Ethics Statement

This study was approved by the Joint Ethics Committee of the Central South University Health Authority, and was performed according to the ethical standards of the Declaration of Helsinki. All animal studies were approved and performed according to the guidelines of the Institutional Animal Care and Use Committee (IACUC) of Central South University.

## Author Contributions

MW, SX, and LL designed the manuscript. XS, YZ, CL, PL, SC, BS, YL and YS collected the samples and performed the experiments. JF and JL analyzed and interpreted the data. CZ and MW were the major contributors in writing the manuscript. All authors read and approved the final manuscript.

### Conflict of Interest

The authors declare that the research was conducted in the absence of any commercial or financial relationships that could be construed as a potential conflict of interest.

## Supplementary Material

Figure S1 The effect of LRRC4 ectopic expression on EOC cells proliferation and invasion. **(A)** LRRC4 protein expression was analyzed in five epithelial ovarian cancer cell lines by the use of Western blotting and RT-qPCR. **(B)** RT-qPCR and **(C)** Western blotting analyses were used to detect the expression of LRRC4 in SKOV3 with stable overexpression LRRC4 or control vector through lentivirus infection. **(D)** EDU and **(E)** Transwell assays were used to detect cell proliferation and invasion in SKOV3 cells with stable overexpression LRRC4 or control vector by through lentivirus infection.

Figure S2 The expression of LRRC4 in the intraperitoneal xenografted mouse model.

## Abbreviations

EOC: Epithelial Ovarian Cancer
HGSC: High-grade serous ovarian cancer
LMSC: Low malignant serous ovarian cancer
LRRC4: Leucine-rich repeat-containing protein 4.
