# Supplementary figures and images for: LRRC4 Suppresses E-Cadherin-Dependent Collective Cell Invasion and Metastasis in Epithelial Ovarian Cancer
Source: Front Oncol. 2020 Feb 14;10:144. doi: 10.3389/fonc.2020.00144 (PMC7033568; doi:10.3389/fonc.2020.00144)

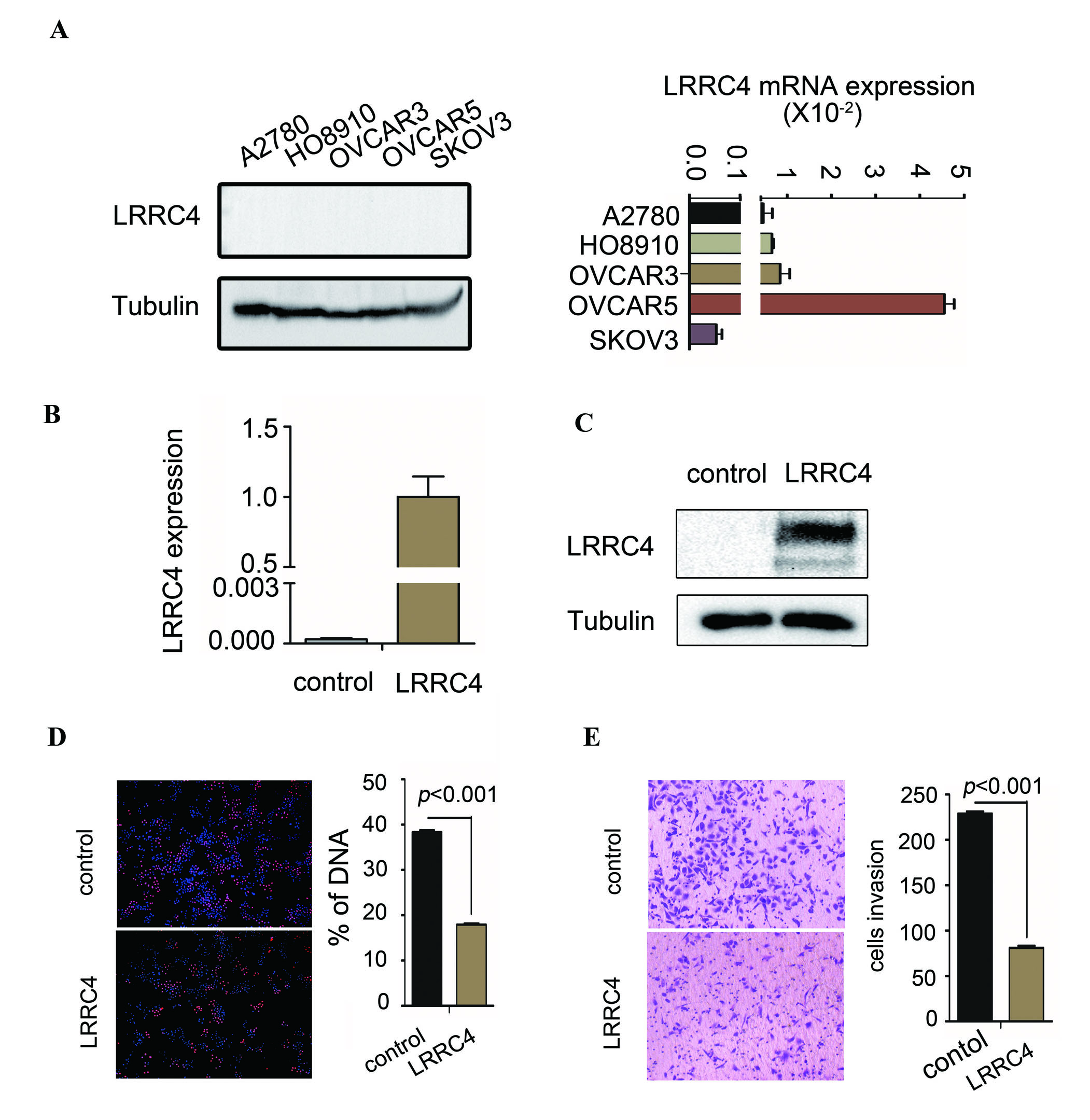

Supplement: Figure S1 — The effect of LRRC4 ectopic expression on EOC cells proliferation and invasion. (A) LRRC4 protein expression was analyzed in five epithelial ovarian cancer cell lines by the use of Western blotting and RT-qPCR. (B) RT-qPCR and (C) Western blotting analyses were used to detect the expression of LRRC4 in SKOV3 with stable overexpression LRRC4 or control vector through lentivirus infection. (D) EDU and (E) Transwell assays were used to detect cell proliferation and invasion in SKOV3 cells with stable overexpression LRRC4 or control vector by through lentivirus infection. [file Image_1.JPEG]

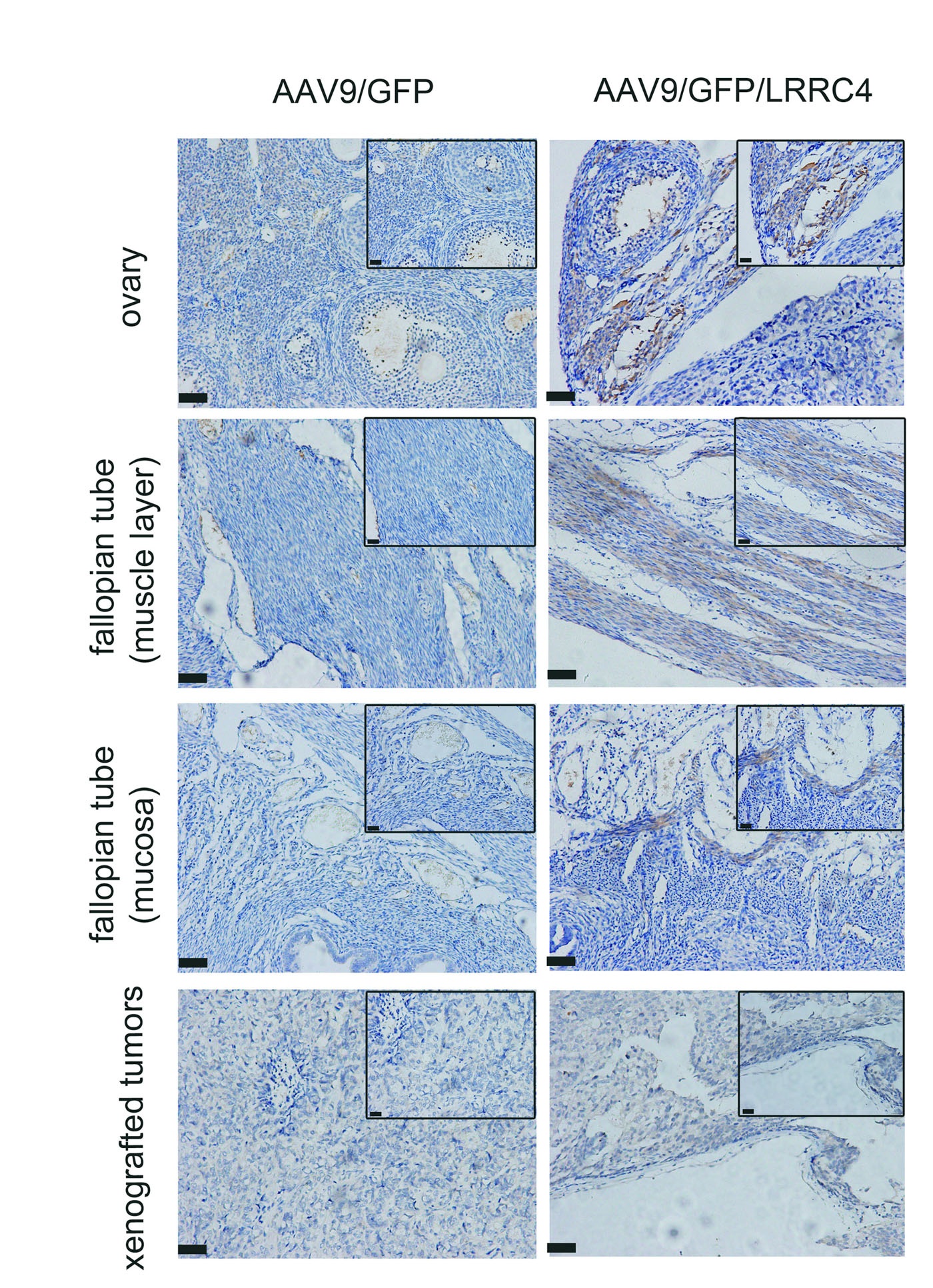

Supplement: Figure S2 — The expression of LRRC4 in the intraperitoneal xenografted mouse model. [file Image_2.JPEG]
